# Bis(5-amino-4-amino­carbonyl-1*H*-imid­azol-3-ium) (5-amino-4-amino­carbonyl-1*H*-imidazol-3-ium-κ*O*)-di-μ-chlorido-hepta­chlorido-dibismuth(III) mono­hydrate

**DOI:** 10.1107/S1600536808009367

**Published:** 2008-04-10

**Authors:** Lu-Ping Lv, Lian-Jun He, Wei-Wei Li, Wen-Bo Yu, Xian-Chao Hu

**Affiliations:** a Department of Chemical Engineering, Hangzhou Vocational and Technical College, Hangzhou 310018, People’s Republic of China; b Research Center for Analysis and Measurement, Zhejiang University of Technology, Hangzhou 310014, People’s Republic of China

## Abstract

The title compound, (C_4_H_7_N_4_O)_2_[Bi_2_Cl_9_(C_4_H_7_N_4_O)]·H_2_O, was prepared by the reaction of bis­muth trichloride and 5-amino-1*H*-imidazole-4-carboxamide in a dilute HCl medium. The asymmetric unit contains two 5-amino-4-amino­carbonyl-1*H*-imidazol-3-ium cations, one edge-shared non-centrosymmetric biocta­hedral [Bi_2_C1_9_(C_4_H_7_N_4_O)]^2−^ dianion and a water mol­ecule. In the dianion, the planar 5-amino-4-amino­carbonyl-1*H*-imidazol-3-ium ligand occupies an equatorial site and is inclined at an angle of 75.7 (2)° to the Bi_2_(μ-C1)_2_ plane. The salt forms a three-dimensional network arising from hydrogen bonds between cations, anions and water mol­ecules.

## Related literature

For general background, see: Turel *et al.* (1998 ▶); Goforth *et al.* (2004 ▶). For related structures, see: Fu *et al.* (2005 ▶); Wu *et al.* (2005 ▶); Kyriakidis *et al.*, (1990 ▶). For a description of the Cambridge Structural Database, see: Allen (2002 ▶).
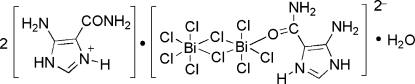


## Experimental

### 

#### Crystal data

(C_4_H_7_N_4_O)_2_[Bi_2_Cl_9_(C_4_H_7_N_4_O)]·H_2_O
*M* *_r_* = 1136.43
Triclinic, 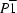

*a* = 11.3365 (5) Å
*b* = 12.2486 (6) Å
*c* = 12.7919 (6) Å
α = 74.433 (3)°
β = 65.939 (3)°
γ = 75.397 (3)°
*V* = 1541.71 (12) Å^3^
*Z* = 2
Mo *K*α radiation
μ = 12.22 mm^−1^
*T* = 123 (2) K
0.25 × 0.22 × 0.20 mm

#### Data collection

Rigaku Mercury diffractometer
Absorption correction: multi-scan (Jacobson, 1998 ▶) *T*_min_ = 0.150, *T*_max_ = 0.194 (expected range = 0.067–0.087)
18217 measured reflections
5695 independent reflections
4988 reflections with *I* > 2σ(*I*)
*R*_int_ = 0.063

#### Refinement

*R*[*F* ^2^ > 2σ(*F* ^2^)] = 0.074
*wR*(*F* ^2^) = 0.223
*S* = 1.10
5695 reflections
359 parameters
2 restraints
H atoms treated by a mixture of independent and constrained refinement
Δρ_max_ = 5.85 e Å^−3^
Δρ_min_ = −4.41 e Å^−3^

### 

Data collection: *CrystalClear* (Rigaku, 2001 ▶); cell refinement: *CrystalClear*; data reduction: *CrystalStructure* (Rigaku/MSC, 2004 ▶); program(s) used to solve structure: *SHELXS97* (Sheldrick, 2008 ▶); program(s) used to refine structure: *SHELXL97* (Sheldrick, 2008 ▶); molecular graphics: *SHELXTL* (Sheldrick, 2008 ▶); software used to prepare material for publication: *SHELXTL*.

## Supplementary Material

Crystal structure: contains datablocks I, global. DOI: 10.1107/S1600536808009367/ci2573sup1.cif

Structure factors: contains datablocks I. DOI: 10.1107/S1600536808009367/ci2573Isup2.hkl

Additional supplementary materials:  crystallographic information; 3D view; checkCIF report

## Figures and Tables

**Table 1:** Selected bond lengths (Å)

Bi1—O1	2.464 (10)
Bi1—Cl1	2.543 (3)
Bi1—Cl2	2.589 (4)
Bi1—Cl3	2.601 (4)
Bi1—Cl4	2.872 (4)
Bi1—Cl5	2.921 (4)
Bi2—Cl7	2.535 (3)
Bi2—Cl8	2.606 (4)
Bi2—Cl6	2.676 (4)
Bi2—Cl9	2.725 (4)
Bi2—Cl5	2.859 (4)
Bi2—Cl4	2.928 (4)

**Table 2:** Hydrogen-bond geometry (Å, °)

*D*—H⋯*A*	*D*—H	H⋯*A*	*D*⋯*A*	*D*—H⋯*A*
O4—H4*C*⋯Cl8^i^	0.82 (12)	2.44 (14)	3.215 (11)	158 (16)
O4—H4*D*⋯Cl1	0.83 (12)	2.38 (13)	3.190 (12)	168 (17)
N1—H1⋯Cl6^ii^	0.88	2.36	3.226 (12)	169
N2—H2⋯Cl9^iii^	0.88	2.30	3.166 (11)	170
N3—H3*A*⋯O1	0.88	2.33	2.869 (17)	120
N3—H3*A*⋯Cl1	0.88	2.82	3.649 (15)	157
N3—H3*B*⋯Cl8^iv^	0.88	2.71	3.451 (14)	142
N5—H5⋯O4^v^	0.88	1.87	2.725 (16)	163
N6—H6⋯Cl3^vi^	0.88	2.40	3.230 (12)	158
N7—H7*A*⋯Cl5^vi^	0.88	2.53	3.358 (16)	156
N7—H7*B*⋯O2	0.88	2.24	2.802 (18)	121
N8—H8*A*⋯O2^v^	0.88	1.96	2.821 (15)	166
N8—H8*B*⋯O4^v^	0.88	2.04	2.905 (18)	168
N9—H9⋯Cl2	0.88	2.63	3.348 (13)	140
N10—H10⋯Cl4^vii^	0.88	2.37	3.249 (12)	175
N11—H11*A*⋯O3	0.88	2.30	2.850 (18)	120
N11—H11*A*⋯Cl5^vi^	0.88	2.83	3.426 (14)	127
N11—H11*B*⋯Cl2	0.88	2.70	3.447 (15)	143
N12—H12*A*⋯Cl9^viii^	0.88	2.45	3.315 (13)	168
N12—H12*B*⋯Cl4^vii^	0.88	2.65	3.529 (15)	177

Symmetry codes: (i) 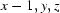; (ii) 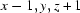; (iii) 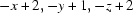; (iv) 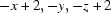; (v) 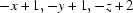; (vi) 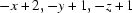; (vii) 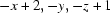; (viii) 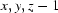.

**Fig. 1.**
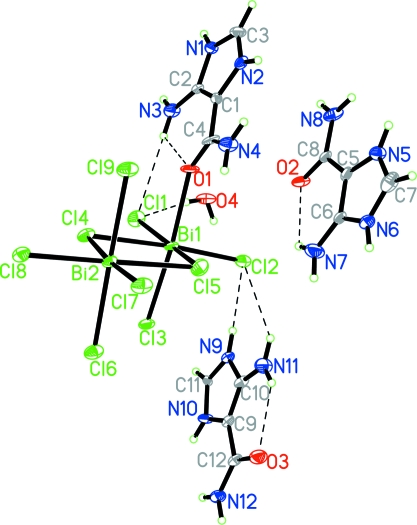
The asymmetric unit of the title compound. Displacement ellipsoids are drawn at the 40% probability level.

**Fig. 2.**
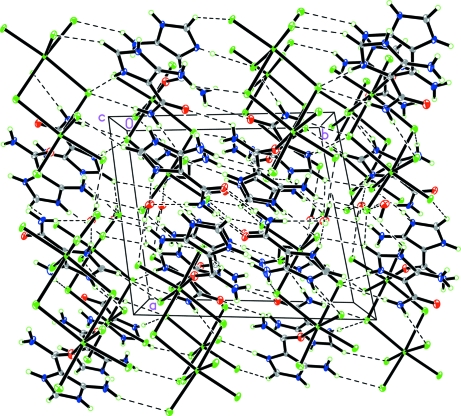
The crystal packing of the title compound, viewed approximately down the c axis. Dashed lines indicate intermolecular hydrogen bonds.

## supplementary crystallographic information

### Comment

Bismuth trihalides have an extensive coordination chemistry as a result of the Lewis acidity of the group 15 element centre. Recently, there is increasing interest in halobismuthate(III) compounds, due to their anti-ulcer activity (Turel *et al.*, 1998) and their unique optical and electronic properties, including nonlinear optical activity, luminescence and semiconductivity (Goforth *et al.*, 2004). We report here the crystal structure of the title organic–inorganic hybrid complex.

The asymmetric unit of the title compound contains two 5-amino-4-aminocarbonyl-1*H*-imidazol-3-ium (C_4_H_7_N_4_O^+^) cations, an edge-shared bi-octahedral dianion {[Bi_2_C1_9_(C_4_H_7_N_4_O)]^2-^} and a water molecule. The dianion of the title compound is non-centrosymmetric compared to large number of centrosymmetric decanchlorobismuthates that have been crystallographically verified, as exemplified by (C_5_H_14_N_2_)_2_[Bi_2_Cl_10_].2H_2_O (Fu *et al.*, 2005) and (C_4_H_12_N_2_)_2_[Bi_2_Cl_10_].3H_2_O (Wu *et al.*, 2005). A search of the Cambridge Structural Database (Version 5.29, January 2008; Allen, 2002) yielded no hits for noncentrosymmetric octanchlorobismuthates.

In the noncentrosymmetric edge-shared bi-octahedral dianion, the planar 5-amino-4-aminocarbonyl-1*H*-imidazol-3-ium ligand occupied a octahedral terminal site, inclined at angle of 75.7 (2)° to the Bi_2_(µ-C1)_2_ plane. Atoms Cl1, Cl2, Cl4, Cl5, Cl7, Cl8, Bi1 and Bi2 are coplaner, with an r.m.s. deviation of 0.120 Å. The Bi—O distance of 2.464 (10) Å is slightly longer compared to the reported value of of 2.424 (10) Å (Kyriakidis *et al.*, 1990). The Bi—Cl distances (Table 1) lie in the range 2.535 (3) Å-2.928 (4) Å, with the Bi—C1 distances involving the bridging C1 atoms being longer (2.859 (4) Å-2.928 (4) Å). None of the interbond angles deviate significantly (>10°) from idealized octahedral angles.

The N—H···O, N—H···Cl and O—H···Cl hydrogen bonds (Table 2) link the constituent ions and water molecules into a three-dimensional network (Fig.2).

### Experimental

The title compound was prepared by the reaction of bismuth trichloride (0.500 g, 1.59 mmol) and 5-amino-4-carboxamide-1H-imidazole (0.601,4.9 mmol) in a hydrochloric acid medium. Yellow crystals suitable for X-ray analysis were obtained by slow evaporation of an ethanol solution of the title compound at room temperature.

### Refinement

Water H atoms were located in a difference map and their positional parameters were refined with a O-H distance restraint of 0.85 (3) Å. All other H atoms were placed at calculated positions (N-H = 0.88 Å and C-H = 0.95 Å) and refined using a riding model, with *U*_iso_(H) = 1.2*U*_eq_(C,N,O). The highest residual density peak is located 0.89 Å from atom Bi1 and the deepest hole is located 0.77 Å from atom Bi2.

### Crystal data

**Table:**

(C_4_H_7_N_4_O)_2_[Bi_2_Cl_9_(C_4_H_7_N_4_O)]·H_2_O	*Z* = 2
*M**_r_* = 1136.43	*F*_000_ = 1060
Triclinic, *P*1	*D*_x_ = 2.448 Mg m^−^^3^
Hall symbol: -P 1	Mo *K*α radiation λ = 0.71073 Å
*a* = 11.3365 (5) Å	Cell parameters from 5695 reflections
*b* = 12.2486 (6) Å	θ = 1.8–25.5º
*c* = 12.7919 (6) Å	µ = 12.22 mm^−^^1^
α = 74.433 (3)º	*T* = 123 (2) K
β = 65.939 (3)º	Block, yellow
γ = 75.397 (3)º	0.25 × 0.22 × 0.20 mm
*V* = 1541.71 (12) Å^3^	

### Data collection

**Table:**

Rigaku Mercury diffractometer	5695 independent reflections
Radiation source: fine-focus sealed tube	4988 reflections with *I* > 2σ(*I*)
Monochromator: graphite	*R*_int_ = 0.063
Detector resolution: 7.31 pixels mm^-1^	θ_max_ = 25.5º
*T* = 123(2) K	θ_min_ = 1.8º
ω scans	*h* = −13→13
Absorption correction: multi-scan(Jacobson, 1998)	*k* = −14→12
*T*_min_ = 0.150, *T*_max_ = 0.194	*l* = −15→15
18217 measured reflections	

### Refinement

**Table:**

Refinement on *F*^2^	Secondary atom site location: difference Fourier map
Least-squares matrix: full	Hydrogen site location: inferred from neighbouring sites
*R*[*F*^2^ > 2σ(*F*^2^)] = 0.075	H atoms treated by a mixture of independent and constrained refinement
*wR*(*F*^2^) = 0.223	*w* = 1/[σ^2^(*F*_o_^2^) + (0.1568*P*)^2^ + 11.6845*P*] where *P* = (*F*_o_^2^ + 2*F*_c_^2^)/3
*S* = 1.10	(Δ/σ)_max_ = 0.001
5695 reflections	Δρ_max_ = 5.85 e Å^−^^3^
359 parameters	Δρ_min_ = −4.41 e Å^−^^3^
2 restraints	Extinction correction: SHELXL97 (Sheldrick, 2008), Fc^*^=kFc[1+0.001xFc^2^λ^3^/sin(2θ)]^-1/4^
Primary atom site location: structure-invariant direct methods	Extinction coefficient: 0.0044 (6)

### Special details

**Table:**

Geometry. All e.s.d.'s (except the e.s.d. in the dihedral angle between two l.s. planes) are estimated using the full covariance matrix. The cell e.s.d.'s are taken into account individually in the estimation of e.s.d.'s in distances, angles and torsion angles; correlations between e.s.d.'s in cell parameters are only used when they are defined by crystal symmetry. An approximate (isotropic) treatment of cell e.s.d.'s is used for estimating e.s.d.'s involving l.s. planes.
Refinement. Refinement of *F*^2^ against ALL reflections. The weighted *R*-factor *wR* and goodness of fit *S* are based on *F*^2^, conventional *R*-factors *R* are based on *F*, with *F* set to zero for negative *F*^2^. The threshold expression of *F*^2^ > σ(*F*^2^) is used only for calculating *R*-factors(gt) *etc*. and is not relevant to the choice of reflections for refinement. *R*-factors based on *F*^2^ are statistically about twice as large as those based on *F*, and *R*-factors based on ALL data will be even larger.

### Fractional atomic coordinates and isotropic or equivalent isotropic displacement parameters (Å^2^)

**Table:**

	*x*	*y*	*z*	*U*_iso_*/*U*_eq_	
Bi1	0.88808 (4)	0.20130 (4)	0.77380 (4)	0.0190 (2)	
Bi2	1.29373 (4)	0.22332 (4)	0.68713 (4)	0.0197 (2)	
Cl1	0.7650 (4)	0.0403 (3)	0.9119 (3)	0.0322 (8)	
Cl2	0.6905 (4)	0.3183 (3)	0.7184 (3)	0.0327 (8)	
Cl3	0.9668 (4)	0.0975 (3)	0.5986 (3)	0.0260 (8)	
Cl4	1.1107 (4)	0.0608 (3)	0.8230 (3)	0.0279 (8)	
Cl5	1.0678 (4)	0.3675 (3)	0.6497 (3)	0.0351 (9)	
Cl6	1.3711 (4)	0.1462 (3)	0.4887 (3)	0.0303 (8)	
Cl7	1.4310 (4)	0.3802 (3)	0.5692 (3)	0.0310 (8)	
Cl8	1.4877 (4)	0.0783 (3)	0.7303 (3)	0.0297 (8)	
Cl9	1.2315 (4)	0.3140 (3)	0.8792 (3)	0.0332 (9)	
O1	0.8130 (11)	0.2826 (10)	0.9511 (9)	0.032 (2)	
O2	0.6215 (10)	0.5277 (8)	0.8614 (8)	0.028 (2)	
O3	0.9864 (12)	0.3596 (10)	0.1801 (10)	0.037 (3)	
O4	0.4595 (11)	0.1325 (10)	0.9725 (9)	0.036 (2)	
H4C	0.448 (17)	0.134 (15)	0.913 (9)	0.043*	
H4D	0.535 (6)	0.100 (14)	0.965 (16)	0.043*	
N1	0.5550 (11)	0.3189 (10)	1.2864 (10)	0.023 (2)	
H1	0.5002	0.2801	1.3472	0.027*	
N2	0.6655 (10)	0.4515 (9)	1.1772 (9)	0.021 (2)	
H2	0.6968	0.5165	1.1522	0.025*	
N3	0.6306 (14)	0.1787 (11)	1.1651 (12)	0.030 (3)	
H3A	0.6824	0.1572	1.0986	0.036*	
H3B	0.5778	0.1332	1.2196	0.036*	
N4	0.8721 (12)	0.4479 (11)	0.9441 (11)	0.032 (3)	
H4A	0.9323	0.4469	0.8739	0.038*	
H4B	0.8592	0.5043	0.9799	0.038*	
N5	0.7091 (11)	0.8051 (10)	0.8213 (10)	0.026 (3)	
H5	0.6647	0.8364	0.8836	0.031*	
N6	0.8406 (12)	0.7822 (10)	0.6487 (10)	0.026 (3)	
H6	0.8993	0.7956	0.5778	0.032*	
N7	0.8068 (18)	0.5992 (14)	0.6422 (13)	0.049 (4)	
H7A	0.8624	0.6028	0.5697	0.059*	
H7B	0.7669	0.5390	0.6781	0.059*	
N8	0.5411 (11)	0.6387 (11)	1.0021 (10)	0.029 (3)	
H8A	0.4925	0.5889	1.0546	0.035*	
H8B	0.5414	0.7033	1.0198	0.035*	
N9	0.7241 (11)	0.2073 (10)	0.4935 (10)	0.024 (2)	
H9	0.6776	0.2215	0.5642	0.029*	
N10	0.8048 (11)	0.1235 (10)	0.3465 (10)	0.023 (2)	
H10	0.8222	0.0725	0.3029	0.028*	
N11	0.8338 (14)	0.3714 (11)	0.4186 (12)	0.036 (3)	
H11A	0.8904	0.4112	0.3607	0.043*	
H11B	0.7929	0.3944	0.4859	0.043*	
N12	0.9985 (13)	0.2033 (12)	0.1141 (11)	0.030 (3)	
H12A	1.0563	0.2282	0.0459	0.037*	
H12B	0.9727	0.1373	0.1270	0.037*	
C1	0.7034 (12)	0.3655 (11)	1.1141 (11)	0.020 (3)	
C2	0.6319 (12)	0.2801 (11)	1.1833 (11)	0.019 (3)	
C3	0.5752 (16)	0.4227 (14)	1.2810 (13)	0.030 (3)	
H3	0.5331	0.4677	1.3402	0.036*	
C4	0.7999 (13)	0.3635 (13)	0.9963 (11)	0.025 (3)	
C5	0.7001 (12)	0.7001 (11)	0.8102 (12)	0.021 (3)	
C6	0.7828 (13)	0.6855 (13)	0.6981 (12)	0.023 (3)	
C7	0.7944 (16)	0.8524 (17)	0.7245 (16)	0.036 (4)	
H7	0.8190	0.9250	0.7110	0.043*	
C8	0.6157 (13)	0.6159 (12)	0.8940 (11)	0.022 (3)	
C9	0.8589 (13)	0.2245 (12)	0.3115 (12)	0.022 (3)	
C10	0.8098 (13)	0.2759 (12)	0.4041 (12)	0.023 (3)	
C11	0.7240 (12)	0.1182 (10)	0.4551 (11)	0.020 (3)	
H11	0.6732	0.0589	0.4993	0.024*	
C12	0.9513 (13)	0.2642 (12)	0.1958 (12)	0.022 (3)	

### Atomic displacement parameters (Å^2^)

**Table:**

	*U*^11^	*U*^22^	*U*^33^	*U*^12^	*U*^13^	*U*^23^
Bi1	0.0240 (3)	0.0227 (4)	0.0089 (3)	−0.0076 (2)	−0.0030 (2)	−0.0021 (2)
Bi2	0.0241 (3)	0.0198 (4)	0.0132 (3)	−0.0090 (2)	−0.0041 (2)	0.0009 (2)
Cl1	0.0335 (17)	0.0309 (19)	0.0273 (19)	−0.0177 (15)	−0.0048 (15)	0.0037 (15)
Cl2	0.0371 (18)	0.039 (2)	0.0231 (18)	0.0031 (16)	−0.0130 (15)	−0.0127 (15)
Cl3	0.0366 (17)	0.0275 (18)	0.0119 (16)	−0.0086 (14)	−0.0055 (13)	−0.0029 (13)
Cl4	0.0378 (19)	0.0293 (19)	0.0177 (17)	−0.0132 (15)	−0.0102 (14)	0.0013 (14)
Cl5	0.041 (2)	0.031 (2)	0.0256 (19)	−0.0096 (16)	−0.0075 (16)	0.0039 (16)
Cl6	0.047 (2)	0.0273 (19)	0.0214 (18)	−0.0161 (16)	−0.0126 (16)	−0.0019 (15)
Cl7	0.0423 (19)	0.0284 (19)	0.0200 (18)	−0.0222 (15)	−0.0033 (15)	0.0016 (14)
Cl8	0.0342 (17)	0.0319 (19)	0.0198 (17)	−0.0073 (15)	−0.0083 (14)	−0.0003 (14)
Cl9	0.043 (2)	0.035 (2)	0.0185 (18)	−0.0209 (16)	−0.0029 (15)	−0.0004 (15)
O1	0.050 (6)	0.035 (6)	0.012 (5)	−0.008 (5)	−0.008 (5)	−0.012 (4)
O2	0.042 (5)	0.032 (6)	0.013 (5)	−0.018 (4)	−0.008 (4)	−0.003 (4)
O3	0.049 (6)	0.032 (6)	0.022 (6)	−0.011 (5)	−0.004 (5)	−0.002 (5)
O4	0.039 (6)	0.052 (7)	0.018 (5)	−0.015 (5)	−0.004 (5)	−0.014 (5)
N1	0.035 (6)	0.015 (6)	0.014 (6)	−0.007 (5)	−0.004 (5)	0.001 (5)
N2	0.030 (5)	0.017 (6)	0.018 (6)	−0.010 (5)	−0.011 (5)	0.005 (4)
N3	0.048 (8)	0.020 (7)	0.019 (7)	−0.016 (6)	−0.006 (6)	0.003 (5)
N4	0.042 (7)	0.030 (7)	0.019 (6)	−0.020 (6)	−0.001 (5)	0.000 (5)
N5	0.034 (6)	0.025 (6)	0.021 (6)	−0.011 (5)	−0.008 (5)	−0.003 (5)
N6	0.032 (6)	0.024 (7)	0.015 (6)	−0.008 (5)	−0.004 (5)	0.002 (5)
N7	0.074 (11)	0.032 (8)	0.021 (8)	−0.008 (8)	0.003 (7)	−0.007 (6)
N8	0.034 (6)	0.041 (7)	0.016 (6)	−0.024 (6)	−0.007 (5)	0.003 (5)
N9	0.030 (6)	0.024 (6)	0.014 (6)	−0.004 (5)	−0.006 (5)	0.001 (5)
N10	0.031 (6)	0.026 (6)	0.013 (6)	−0.011 (5)	−0.002 (5)	−0.007 (5)
N11	0.053 (8)	0.024 (7)	0.028 (7)	−0.014 (6)	−0.008 (6)	−0.005 (6)
N12	0.033 (6)	0.029 (7)	0.017 (7)	−0.008 (5)	0.001 (5)	0.001 (5)
C1	0.024 (6)	0.017 (6)	0.012 (6)	0.001 (5)	−0.003 (5)	−0.002 (5)
C2	0.024 (6)	0.021 (7)	0.012 (6)	−0.002 (5)	−0.009 (5)	−0.001 (5)
C3	0.047 (9)	0.027 (8)	0.014 (7)	−0.006 (7)	−0.008 (6)	−0.006 (6)
C4	0.031 (7)	0.037 (8)	0.008 (6)	−0.013 (6)	−0.007 (5)	0.001 (6)
C5	0.023 (6)	0.018 (7)	0.017 (7)	−0.002 (5)	−0.009 (5)	0.003 (5)
C6	0.024 (6)	0.023 (7)	0.013 (7)	0.001 (5)	−0.004 (5)	0.003 (5)
C7	0.029 (8)	0.040 (10)	0.034 (10)	−0.013 (7)	−0.013 (7)	0.009 (8)
C8	0.023 (6)	0.025 (8)	0.011 (6)	0.001 (6)	−0.006 (5)	0.003 (6)
C9	0.027 (6)	0.021 (7)	0.016 (7)	−0.003 (5)	−0.009 (6)	0.001 (6)
C10	0.027 (6)	0.027 (7)	0.016 (7)	−0.010 (6)	−0.010 (6)	0.001 (6)
C11	0.031 (6)	0.007 (6)	0.013 (6)	0.000 (5)	0.000 (5)	−0.001 (5)
C12	0.024 (6)	0.024 (7)	0.015 (7)	−0.009 (5)	−0.007 (5)	0.003 (6)

### Geometric parameters (Å, °)

**Table:**

Bi1—O1	2.464 (10)	N5—H5	0.88
Bi1—Cl1	2.543 (3)	N6—C7	1.33 (2)
Bi1—Cl2	2.589 (4)	N6—C6	1.372 (19)
Bi1—Cl3	2.601 (4)	N6—H6	0.88
Bi1—Cl4	2.872 (4)	N7—C6	1.34 (2)
Bi1—Cl5	2.921 (4)	N7—H7A	0.88
Bi2—Cl7	2.535 (3)	N7—H7B	0.88
Bi2—Cl8	2.606 (4)	N8—C8	1.356 (18)
Bi2—Cl6	2.676 (4)	N8—H8A	0.88
Bi2—Cl9	2.725 (4)	N8—H8B	0.88
Bi2—Cl5	2.859 (4)	N9—C11	1.312 (17)
Bi2—Cl4	2.928 (4)	N9—C10	1.395 (17)
O1—C4	1.226 (18)	N9—H9	0.88
O2—C8	1.237 (17)	N10—C11	1.311 (16)
O3—C12	1.267 (18)	N10—C9	1.403 (18)
O4—H4C	0.82 (12)	N10—H10	0.88
O4—H4D	0.83 (12)	N11—C10	1.340 (19)
N1—C3	1.33 (2)	N11—H11A	0.88
N1—C2	1.383 (18)	N11—H11B	0.88
N1—H1	0.88	N12—C12	1.30 (2)
N2—C3	1.326 (19)	N12—H12A	0.88
N2—C1	1.375 (17)	N12—H12B	0.88
N2—H2	0.88	C1—C2	1.371 (18)
N3—C2	1.328 (19)	C1—C4	1.459 (18)
N3—H3A	0.88	C3—H3	0.95
N3—H3B	0.88	C5—C6	1.39 (2)
N4—C4	1.340 (18)	C5—C8	1.482 (19)
N4—H4A	0.88	C7—H7	0.95
N4—H4B	0.88	C9—C10	1.34 (2)
N5—C7	1.31 (2)	C9—C12	1.459 (19)
N5—C5	1.364 (18)	C11—H11	0.95
			
O1—Bi1—Cl1	83.9 (3)	C6—N7—H7B	120.0
O1—Bi1—Cl2	92.6 (3)	H7A—N7—H7B	120.0
Cl1—Bi1—Cl2	94.34 (13)	C8—N8—H8A	120.0
O1—Bi1—Cl3	174.9 (3)	C8—N8—H8B	120.0
Cl1—Bi1—Cl3	91.66 (12)	H8A—N8—H8B	120.0
Cl2—Bi1—Cl3	90.18 (12)	C11—N9—C10	108.9 (11)
O1—Bi1—Cl4	91.6 (3)	C11—N9—H9	125.5
Cl1—Bi1—Cl4	84.94 (12)	C10—N9—H9	125.5
Cl2—Bi1—Cl4	175.67 (11)	C11—N10—C9	108.3 (11)
Cl3—Bi1—Cl4	85.58 (11)	C11—N10—H10	125.9
O1—Bi1—Cl5	89.9 (3)	C9—N10—H10	125.9
Cl1—Bi1—Cl5	166.27 (13)	C10—N11—H11A	120.0
Cl2—Bi1—Cl5	98.13 (12)	C10—N11—H11B	120.0
Cl3—Bi1—Cl5	94.01 (12)	H11A—N11—H11B	120.0
Cl4—Bi1—Cl5	83.05 (11)	C12—N12—H12A	120.0
Cl7—Bi2—Cl8	93.94 (13)	C12—N12—H12B	120.0
Cl7—Bi2—Cl6	86.98 (12)	H12A—N12—H12B	120.0
Cl8—Bi2—Cl6	87.69 (12)	C2—C1—N2	106.6 (11)
Cl7—Bi2—Cl9	88.05 (12)	C2—C1—C4	125.7 (12)
Cl8—Bi2—Cl9	91.62 (12)	N2—C1—C4	127.6 (12)
Cl6—Bi2—Cl9	174.93 (11)	N3—C2—C1	131.4 (13)
Cl7—Bi2—Cl5	90.85 (13)	N3—C2—N1	123.2 (12)
Cl8—Bi2—Cl5	175.20 (11)	C1—C2—N1	105.4 (12)
Cl6—Bi2—Cl5	92.26 (12)	N2—C3—N1	106.9 (13)
Cl9—Bi2—Cl5	88.84 (13)	N2—C3—H3	126.6
Cl7—Bi2—Cl4	173.86 (12)	N1—C3—H3	126.6
Cl8—Bi2—Cl4	92.07 (11)	O1—C4—N4	123.2 (13)
Cl6—Bi2—Cl4	94.49 (11)	O1—C4—C1	118.0 (12)
Cl9—Bi2—Cl4	90.55 (11)	N4—C4—C1	118.8 (13)
Cl5—Bi2—Cl4	83.14 (11)	N5—C5—C6	106.7 (12)
Bi1—Cl4—Bi2	96.60 (11)	N5—C5—C8	129.6 (13)
Bi2—Cl5—Bi1	97.04 (12)	C6—C5—C8	123.6 (13)
C4—O1—Bi1	149.3 (9)	N7—C6—N6	123.7 (13)
H4C—O4—H4D	108 (19)	N7—C6—C5	130.7 (14)
C3—N1—C2	110.8 (12)	N6—C6—C5	105.5 (13)
C3—N1—H1	124.6	N5—C7—N6	109.0 (16)
C2—N1—H1	124.6	N5—C7—H7	125.5
C3—N2—C1	110.4 (12)	N6—C7—H7	125.5
C3—N2—H2	124.8	O2—C8—N8	124.5 (13)
C1—N2—H2	124.8	O2—C8—C5	117.4 (12)
C2—N3—H3A	120.0	N8—C8—C5	118.0 (13)
C2—N3—H3B	120.0	C10—C9—N10	106.9 (12)
H3A—N3—H3B	120.0	C10—C9—C12	127.4 (13)
C4—N4—H4A	120.0	N10—C9—C12	125.8 (13)
C4—N4—H4B	120.0	N11—C10—C9	130.9 (13)
H4A—N4—H4B	120.0	N11—C10—N9	122.7 (13)
C7—N5—C5	109.4 (14)	C9—C10—N9	106.4 (12)
C7—N5—H5	125.3	N10—C11—N9	109.5 (12)
C5—N5—H5	125.3	N10—C11—H11	125.3
C7—N6—C6	109.3 (13)	N9—C11—H11	125.3
C7—N6—H6	125.3	O3—C12—N12	121.6 (13)
C6—N6—H6	125.3	O3—C12—C9	116.4 (13)
C6—N7—H7A	120.0	N12—C12—C9	121.9 (13)
			
O1—Bi1—Cl4—Bi2	−92.7 (3)	C2—C1—C4—O1	5(2)
Cl1—Bi1—Cl4—Bi2	−176.40 (12)	N2—C1—C4—O1	−174.4 (13)
Cl3—Bi1—Cl4—Bi2	91.54 (12)	C2—C1—C4—N4	−173.0 (13)
Cl5—Bi1—Cl4—Bi2	−3.06 (9)	N2—C1—C4—N4	7(2)
Cl8—Bi2—Cl4—Bi1	−176.47 (11)	C7—N5—C5—C6	−1.8 (16)
Cl6—Bi2—Cl4—Bi1	−88.62 (12)	C7—N5—C5—C8	−178.5 (13)
Cl9—Bi2—Cl4—Bi1	91.89 (12)	C7—N6—C6—N7	178.3 (15)
Cl5—Bi2—Cl4—Bi1	3.12 (10)	C7—N6—C6—C5	−1.2 (15)
Cl7—Bi2—Cl5—Bi1	178.20 (12)	N5—C5—C6—N7	−177.7 (16)
Cl6—Bi2—Cl5—Bi1	91.18 (12)	C8—C5—C6—N7	−1(2)
Cl9—Bi2—Cl5—Bi1	−93.77 (12)	N5—C5—C6—N6	1.8 (14)
Cl4—Bi2—Cl5—Bi1	−3.07 (10)	C8—C5—C6—N6	178.7 (11)
O1—Bi1—Cl5—Bi2	94.8 (3)	C5—N5—C7—N6	1.0 (17)
Cl1—Bi1—Cl5—Bi2	32.2 (6)	C6—N6—C7—N5	0.2 (17)
Cl2—Bi1—Cl5—Bi2	−172.67 (11)	N5—C5—C8—O2	177.0 (13)
Cl3—Bi1—Cl5—Bi2	−81.91 (13)	C6—C5—C8—O2	0.9 (19)
Cl4—Bi1—Cl5—Bi2	3.13 (10)	N5—C5—C8—N8	−5(2)
Cl1—Bi1—O1—C4	−170 (2)	C6—C5—C8—N8	178.5 (12)
Cl2—Bi1—O1—C4	−75.7 (19)	C11—N10—C9—C10	1.7 (15)
Cl4—Bi1—O1—C4	105.4 (19)	C11—N10—C9—C12	−178.5 (12)
Cl5—Bi1—O1—C4	22.4 (19)	N10—C9—C10—N11	176.9 (15)
C3—N2—C1—C2	0.2 (15)	C12—C9—C10—N11	−3(2)
C3—N2—C1—C4	179.9 (13)	N10—C9—C10—N9	−1.4 (14)
N2—C1—C2—N3	−179.8 (14)	C12—C9—C10—N9	178.8 (12)
C4—C1—C2—N3	1(2)	C11—N9—C10—N11	−177.8 (13)
N2—C1—C2—N1	−0.8 (13)	C11—N9—C10—C9	0.7 (14)
C4—C1—C2—N1	179.5 (12)	C9—N10—C11—N9	−1.3 (15)
C3—N1—C2—N3	−179.8 (13)	C10—N9—C11—N10	0.4 (15)
C3—N1—C2—C1	1.2 (15)	C10—C9—C12—O3	−2(2)
C1—N2—C3—N1	0.5 (16)	N10—C9—C12—O3	178.1 (13)
C2—N1—C3—N2	−1.0 (17)	C10—C9—C12—N12	175.8 (14)
Bi1—O1—C4—N4	−22 (3)	N10—C9—C12—N12	−4(2)
Bi1—O1—C4—C1	160.0 (14)		

### Hydrogen-bond geometry (Å, °)

**Table:**

*D*—H···*A*	*D*—H	H···*A*	*D*···*A*	*D*—H···*A*
O4—H4C···Cl8^i^	0.82 (12)	2.44 (14)	3.215 (11)	158 (16)
O4—H4D···Cl1	0.83 (12)	2.38 (13)	3.190 (12)	168 (17)
N1—H1···Cl6^ii^	0.88	2.36	3.226 (12)	169
N2—H2···Cl9^iii^	0.88	2.30	3.166 (11)	170
N3—H3A···O1	0.88	2.33	2.869 (17)	120
N3—H3A···Cl1	0.88	2.82	3.649 (15)	157
N3—H3B···Cl8^iv^	0.88	2.71	3.451 (14)	142
N5—H5···O4^v^	0.88	1.87	2.725 (16)	163
N6—H6···Cl3^vi^	0.88	2.40	3.230 (12)	158
N7—H7A···Cl5^vi^	0.88	2.53	3.358 (16)	156
N7—H7B···O2	0.88	2.24	2.802 (18)	121
N8—H8A···O2^v^	0.88	1.96	2.821 (15)	166
N8—H8B···O4^v^	0.88	2.04	2.905 (18)	168
N9—H9···Cl2	0.88	2.63	3.348 (13)	140
N10—H10···Cl4^vii^	0.88	2.37	3.249 (12)	175
N11—H11A···O3	0.88	2.30	2.850 (18)	120
N11—H11A···Cl5^vi^	0.88	2.83	3.426 (14)	127
N11—H11B···Cl2	0.88	2.70	3.447 (15)	143
N12—H12A···Cl9^viii^	0.88	2.45	3.315 (13)	168
N12—H12B···Cl4^vii^	0.88	2.65	3.529 (15)	177

Symmetry codes: (i) *x*−1, *y*, *z*; (ii) *x*−1, *y*, *z*+1; (iii) −*x*+2, −*y*+1, −*z*+2; (iv) −*x*+2, −*y*, −*z*+2; (v) −*x*+1, −*y*+1, −*z*+2; (vi) −*x*+2, −*y*+1, −*z*+1; (vii) −*x*+2, −*y*, −*z*+1; (viii) *x*, *y*, *z*−1.
